# Risk Factors for Multi-Drug Resistant Pathogens and Failure of Empiric First-Line Therapy in Acute Cholangitis

**DOI:** 10.1371/journal.pone.0169900

**Published:** 2017-01-11

**Authors:** Philipp A. Reuken, Dorian Torres, Michael Baier, Bettina Löffler, Christoph Lübbert, Norman Lippmann, Andreas Stallmach, Tony Bruns

**Affiliations:** 1 Department of Internal Medicine IV (Gastroenterology, Hepatology, and Infectious Diseases), Jena University Hospital, Jena, Germany; 2 Division of Infectious Diseases and Tropical Medicine, Department of Gastroenterology and Rheumatology, Leipzig University Hospital, Leipzig, Germany; 3 Institute of Medical Microbiology, Jena University Hospital, Jena, Germany; 4 Institute for Medical Microbiology and Epidemiology of Infectious Diseases, Leipzig University Hospital, Leipzig, Germany; 5 The Integrated Research and Treatment Center for Sepsis Control and Care (CSCC), Jena University Hospital, Jena, Germany; Seconda Universita degli Studi di Napoli, ITALY

## Abstract

**Background:**

Acute cholangitis (AC) requires the immediate initiation of antibiotic therapy in addition to treatment for biliary obstruction. Against a background of an increasing prevalence of multi-drug resistant (MDR) bacteria, the risk factors for the failure of empiric therapy must be defined.

**Methods:**

Using a pathogen-based approach, 1764 isolates from positive bile duct cultures were retrospectively analyzed to characterize the respective pathogen spectra in two German tertiary centers. Using a patient-based approach, the clinical and laboratory data for 83 patients with AC were assessed to identify risk factors for AC with pathogens resistant to the applied empiric therapy.

**Results:**

Bile cultures were predominantly polymicrobial, and empiric antibiotic therapies did not cover the full biliary pathogen spectrum in 78% of cases. MDR bacteria were isolated from the bile of 24/83 (29%) patients. The univariate risk factors for biliary MDR bacteria were male sex, nosocomial AC, prior antibiotic exposure and prior biliary stenting, of which biliary stenting was the only independent risk factor according to multivariate analysis (OR = 3.8; 95% CI 1.3–11.0, *P* = 0.013). Although there were no significant differences in survival or hospital stay in AC patients with and without detected biliary MDR pathogens, the former more often had a concomitant bloodstream infection (58% vs. 24%; *P* = 0.019), including those involving MDR pathogens or fungi (21% vs. 2%; *P* = 0.007).

**Conclusion:**

Patients with biliary stents who develop AC should receive empiric therapy covering enterococci and extended-spectrum beta-lactamase (ESBL)-producing *Enterobacteriaceae*. These patients are at an increased risk for bloodstream infections by MDR pathogens or fungi.

## Introduction

Acute cholangitis (AC) is a potentially life-threatening bacterial infection of the intra- and/or extrahepatic bile ducts (BD) caused by obstruction of the BD, with stasis and subsequent infection of the bile [1–3]. Common causes of AC are gallstones, BD stenosis in cases of chronic pancreatitis, malignomas and sclerosing cholangitis [1]. The typical symptoms are fever, jaundice and abdominal pain (Charcot’s triad) [4]. Current treatment strategies support a risk-stratified approach based on the revised Tokyo Guidelines [1,5] and generally comprise a combination of antibiotic therapy and early endoscopic resolution of the obstruction [6] because delaying endoscopic treatment often results in persistent organ failure [7].

The pathogens most frequently isolated from bile are Gram-negative *Enterobacteriaceae*, such as *Escherichia coli* and *Klebsiella* spp., as well as *Enterococcus* spp., with a high proportion of polymicrobial cultures observed [8,9]. *Enterococcus* spp. were the biliary pathogens most frequently observed in a recent study from a German tertiary center, with an even higher proportion of *Enterococcus* spp. found in patients who had undergone BD stenting [10]. Bile from healthy individuals is sterile; however, in patients with BD pathologies, bacterial or fungal colonization of the bile without clinical signs of infection may occur [11,12] in up to 100% of patients with biliary stents [13], which can be difficult to discriminate from AC [14].

Because the microbiological identification of pathogens requires time, antibiotic therapy is generally initiated as an empiric therapy. Current guidelines recommend treatment with third-generation cephalosporins (3GC) or a penicillin/beta-lactamase inhibitor-based agent as first-line options for empiric therapy [15,16], initiated as an intravenous infusion. An early switch to oral therapy was found to be non-inferior compared with continuing intravenous infusion [17].

Because microbes and resistance patterns show both regional and temporal variations [18,11,19], the aims of this retrospective study were to (i) characterize the contemporary microbial patterns of BD cultures performed in two German tertiary care centers using a pathogen-based approach and (ii) identify risk factors for AC by pathogens resistant to empiric antibiotic therapies and the associated outcomes using a patient-based approach.

## Methods

### Study design

To characterize the biliary pathogen spectrum and resistance patterns, microbiological data were reviewed to identify all patients with positive bile duct cultures performed at the Jena University Hospital (JUH) between 1996 and 2012 and at the Leipzig University Hospital (LUH) between 2013 and 2015. Cases in which identical pathogen spectra were identified in individual patients during the same hospital stay (copy strains) were excluded from the analysis.

A subset of patients (N = 83) who were admitted between 2006 and 2012 to the JUH were used to identify individual risk factors for acquiring resistant organisms. For this retrospective approach, medical records, including patient files, electronic health records, imaging data, laboratory data, and nursing documentation, were used to retrospectively collect clinical and laboratory data. The following variables were collected from the medical records: age; gender; co-morbidities; medications, including antibiotics and changes in antibiotic therapy; treatment in the intensive care unit (ICU); endoscopic diagnostic procedures and interventions; length of hospital stay and mortality. Nosocomial AC was defined in patients who were hospitalized for 48 h or more before bile fluid sampling because the onset of their symptoms was less well defined. Immunosuppressive therapy was defined as treatment with high-dose prednisolone, azathioprine, anti-TNF-alpha antibodies, calcineurin-inhibitors, such as cyclosporine A or tacrolimus, or cytostatic chemotherapy. The study protocol conformed to the ethical guidelines of the 1975 Declaration of Helsinki and was approved by the internal review board (local ethics committee; registry number 4734-03/16).

### Microbiological sampling

Samples for blood cultures (BC) were collected before or immediately after the initiation of antibiotic treatment by the bedside inoculation of 10 ml of blood into BC bottles (BacT/Alert, bioMérieux, Durham, NC, USA). Bottles were incubated at 37°C until microbial growth was detected or for at least seven days. Bile cultures were obtained via endoscopic retrograde cholangiopancreatography (ERCP) using catheter aspiration. Primary of bile cultures, as well as all subcultures, including those of positive BC cultures, were performed using standard solid media, e.g., Columbia blood agar (SIFIN, Berlin, Germany) for aerobic bacteria and Schaedler agar (Oxoid, Basingstoke, UK) for anaerobic bacteria. The cultivated microorganisms were identified and antibiotic susceptibility testing (MIC) was performed using a VITEK 2 system (bioMérieux, Durham, NC, USA), with the results interpreted according to the DIN- and EUCAST guidelines [20]

### Statistical analysis

Statistical analyses were performed using SPSS 20 (IBM Corp. Released 2013. IBM SPSS Statistics for Windows, Version 22.0. Armonk, NY:IBM Corp) and Prism 5 (GraphPad Software, La Jolla, CA, USA) software. Significant differences were assessed using the nonparametric Mann-Whitney U test or the Kruskal-Wallis test for continuous data or Fisher’s exact test for discrete variables, as appropriate. The risk factors for cholangitis with resistant pathogens were determined by multivariate binary logistic regression, including using significant univariate predictors through stepwise backward elimination. The significance level in two-sided testing was P <0.05, without correction for multiple testing.

## Results

### Overall pathogen spectra and resistance profiles at two tertiary centers

**Fig 1:**
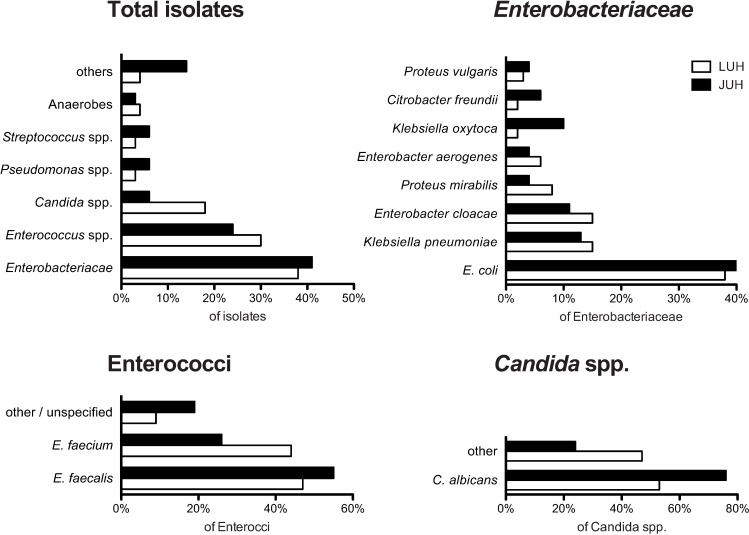
Spectra of biliary pathogens isolated at two tertiary German centers. Proportions of pathogens at the family and species level isolated at the JUH (black bars) and LUH (white bars) overall (upper left), of *Enterobacteriaceae* (upper right), of Enterococci (lower left) and of *Candida* (lower right).

Overall, 1764 isolates were identified from 531 bile cultures (1504 pathogens from 419 cultures at the JUH and 260 pathogens from 112 cultures at the LUH). Seventy-eight percent of cultures were polymicrobial, with a median of two isolated microorganisms (bacteria or fungi) per culture (range: 1–9). On the family level, the most frequently isolated pathogens were *Enterobacteriaceae* (715 isolates; 40.5% of all identified pathogens) and *Enterococcaceae* (440 isolates; 24.9% of all identified pathogens). On the species level, *Escherichia coli* was the most frequently detected pathogen (282 isolates; 16% of all identified pathogens), followed by *Enterococcus faecalis* (236 isolates; 13.4% of all identified pathogens) (Fig 1). *Staphylococcus aureus* was rarely identified (28 isolates, 1.6% of all identified pathogens). Fungi were cultured from 137 of the cultures (7.8%), with all of them found to be *Candida* spp. On the species level, *Candida albicans* was detected in 93 cases (67.9% of all candida isolates) and Candida non-albicans in 44 cases (32.1% of all Candida isolates) (Fig 1).

Contemporary resistance profiles of *E*. *coli* and *Enterococcus* spp. isolates were analyzed using microbiological data between 2006 and 2015 (Table 1). Overall, 27/77 (35.1%) extended-spectrum beta-lactamase (ESBL)-producing *E*. *coli* strains were identified. Quinolone resistance was frequent in the *E*. *coli* isolates (24/77 isolates, 31.2%), whereas resistance to carbapenems was not detected. Isolated enterococci exhibited vancomycin resistance in14/137 cases (Table 1). Ampicillin resistance among enterococci was observed in 45/137 isolates (32.9%), resistance against carbapenems in 53/137 isolates (38.7%) and against linezolid in 2/137 isolates (1.5%, both E. faecalis) (Table 1).

**Table 1:** Resistance profiles of bile isolates of *E*. *coli* and Enterococci 2006–2015.

Antibiotic	E. coli (N = 77)	Enterococci (N = 137)
Ceftriaxone	32.5%	n.a.
Ampicillin/Sulbactam	51.9%	32.9%
Piperacillin/Tazobactam	46.8%	n.a.
Quinolones	31.2%	32.8%*
Carbapenems	0%	38.7%
Vancomycin	n.a.	10.2%
Gentamicin	n.a.	32.2%
Linezolid	n.a.	1.5%

*data available for 58 out of 137 isolates; n.a.: not applicable.

### Resistance to empiric antibiotic therapy

**Fig 2:**
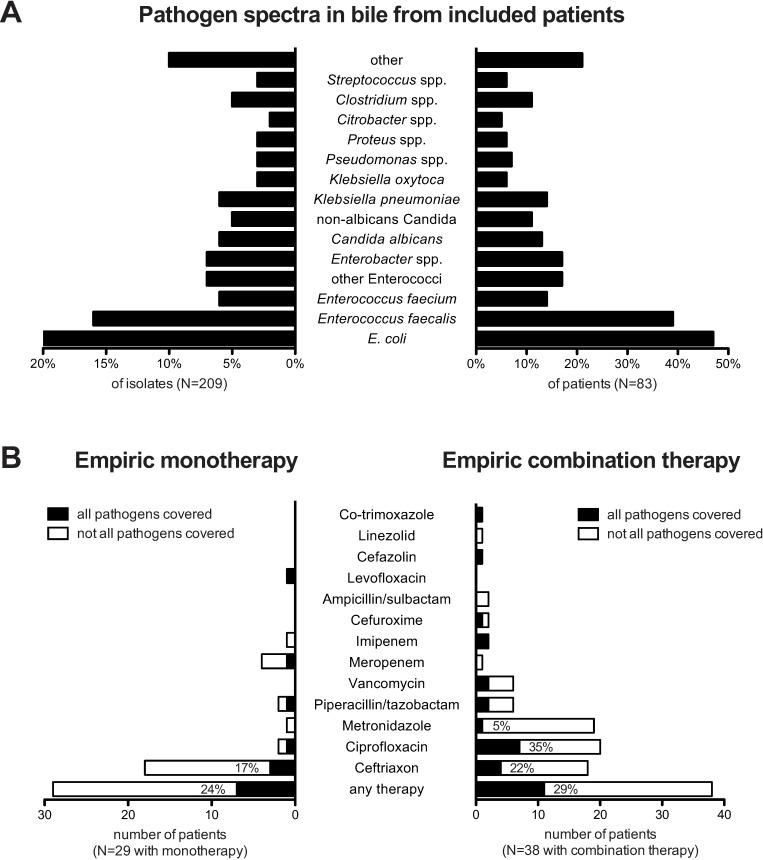
Pathogen spectra and *in vitro* resistance to empiric first-line therapy. 209 pathogens were isolated from bile in 83 episodes of acute cholangitis. (A) The proportion of isolated pathogens is given as the percentage of isolates (left panel) and as the percentage of patients (right panel). (B) *In vitro* susceptibility of isolated pathogens is given for patients treated with empiric monotherapy (left panel) or with empiric combination therapy (therapy). Black bars represent the number of patients, in whom all isolated pathogens were susceptible; the white bars represent the number of patients in whom one or more of the isolated pathogens are resistant against the antibiotic agent. For antibiotic substances, which were used in more than 10 episodes, percentage of fully covered pathogen spectra are shown.

To identify individual risk factors for AC with resistant microorganisms and therapeutic failure, we analyzed the first AC episode of 83 patients seen at the JUH between 2006 and 2012. The pathogen spectrum of the 209 microorganisms, which were isolated from these patients was representative of the overall spectrum described above, and is shown in Fig 2A. Fifty-five (66%) of the patients were male, and the median hospital stay was 8 days. Fifty-two patients (63%) had a previous ERCP, 51 (61%) had a prior papillotomy and 44 (53%) had undergone BD stenting. Fifty-five patients (66%) showed signs of biliary obstruction when microbiological sampling was performed, with choledocholithiasis being the most common cause of obstruction in 46 patients (55% of all patients) (Table 2). Multi-drug resistant (MDR) bacteria were isolated from the bile of 24 (29%) patients, including ESBL-producing *Enterobacteriaceae* (13 patients: 11 *E*. *coli* and 2 *Klebsiella pneumoniae*), vancomycin-resistant *Enterococcus* (VRE) (7 patients), and *Pseudomonas aeruginosa* (4 patients). Among the isolated ESBL-producing *Enterobacteriaceae*, 9 were non-susceptible to at least one agent in at least 3 antimicrobial categories (MDR) and were non-susceptible to at least 1 agent in all but two or fewer categories (XDR) according to the interim definition of the European Centre for Disease Prevention and Control (ECDC) and the Centers for Disease Control and Prevention (CDC) [21]. The identified *Pseudomonas* spp. fulfilled the MDR criteria of the ECDC/CDC.

**Table 2:** Baseline patient characteristics.

	N = 83
Male gender (n)	55 (66.3%)
Admitted to ICU (n)	10 (12%)
Age (years)	69 (60; 76)
Length of stay (days)	8 (6; 18)
WBC (×10^3^/μl)	10.1 (7.6; 13.2)
CRP (mg/l)	85.8 (41.3; 167.6)
ALT (μmol/l×s)	1.02 (0.66; 1.97)
AST (μmol/l×s)	1.11 (0.63; 1.86)
AP (μmol/l×s)	4.26 (2.15; 7.18)
GGT (μmol/l×s)	7.44 (2.73; 12.35)
Bilirubin (μmol/l)	47 (18.5; 103.75)
Prior ERCP	52 (63%)
Prior biliary stent	44 (53%)
Prior papillotomy	51 (61%)
Primary sclerosing cholangitis	5 (6%)
Choledocholithiasis	46 (55%)
Biliary cancer	14 (17%)
Biliary obstruction at ERCP	55 (66%)

Data are presented as absolute numbers and percentages or as median values with first and third quartiles.

Blood cultures were obtained in parallel with bile sampling for 70 of the 83 patients (84%), with positive results obtained for 28 patients (40% of all blood cultures) and negative results obtained for 42 patients (60% of all blood cultures). Two blood cultures had polymicrobial contents (*E*. *coli* and *E*. *faecium*). Fifteen out of the 28 positive cultures displayed identical pathogens in both the blood and bile cultures (54% of all positive blood cultures), including 5 with an identical pathogen and resistance pattern, and in cases of polymicrobial bile culture, 10 cases showed at least one pathogen in the blood culture that was isolated from the bile. The pathogens that were isolated from blood cultures included *E*. *coli* in 14 patients (50% of all positive blood cultures), *Enterococcus* spp. in 5 patients (18% of all positive blood cultures), including *E*. *faecium* (n = 3), *E*. *faecalis* (n = 1) and *E*. *durans* (n = 1), *Klebsiella* spp. for 4 cases (14%), *Staphylococcus hominis* in 3 cases (11%), indicating possible skin contamination, and one case each (4%) of methicillin-resistant *Staphylococcus aureus* (MRSA), *Pseudomonas aeruginosa* and *Streptococcus pneumoniae*. Overall, MDR bacteria were isolated from the blood of 5 patients (7% of patients for whom blood cultures were performed; 6% of all patients) including ESBL-producing *E*. *coli* (3 patients), MRSA (1 patient) and *Pseudomonas aeruginosa* (1 patient). In addition, fungal bloodstream infection by *Candida albicans* was detected in one patient from whom *Candida* was not isolated from the bile.

Twenty-nine patients (35%) received antibiotic monotherapy, 38 (46%) received antibiotic combination therapy, and 16 (19%) did not receive any empiric antibiotic therapy. Ceftriaxone was the antimicrobial substance most frequently used for empiric therapy in 36 of the 83 patients (43%), followed by ciprofloxacin, metronidazole, piperacillin/tazobactam and vancomycin (Fig 2B). According to the in vitro resistance profiles, isolated pathogens were fully susceptible to the empiric monotherapy regimens in 7/29 (24%) patients and to the empiric combination therapy regimens in 11/38 (29%) resulting in overall 65/83 (78%) patients who did not receive an empiric antibiotic before ERCP, covering the isolates from bile. Overall, the initial empiric therapy was changed according to the resistance patterns observed in 40 (60%) of 67 patients. The switching of antibiotic therapy due to *in vitro* resistance by an additionally detected pathogen was frequently observed, occurring in 53% of the patients treated with ceftriaxone (N = 19; 23% of patients) and in 45% of the patients treated with ciprofloxacin (N = 10; 12% of patients) compared with 10% of the patients treated with piperacillin/tazobactam (N = 8; 7% of all patients) and 33% of the patients treated with vancomycin (N = 2; 2% of patients).

### Risk factors for AC with resistant bacteria

Twenty-four (29%) patients with AC had MDR bacteria in bile, including ESBL-producing *Enterobacteriaceae*, VRE, and *Pseudomonas aeruginosa*. AC with MDR bacteria was associated with the male sex (83% vs. 59%; *P* = 0.043), being hospitalized for more than 48 h (58% vs. 32%; P = 0.047), prior biliary stenting (75% vs. 44%, *P* = 0.025) and exposure to antibiotics within 14 days of bile sampling for cultures (42% vs. 17%; *P* = 0.012). In contrast, BD stones, biliary obstruction and previous ERCP were not identified as significant risk factors for MDR pathogens according to univariate analysis (Table 3), and no association with diabetes, chronic lung disease or cirrhosis of the liver was found (data not shown). Expanding the analysis to also include patients with *Candida* in the bile (N = 15), patients with biliary MDR bacteria or *Candida* more often had cholangiocarcinoma (27.0% vs. 8.7%, *P* = 0.039). Because enterococci show intrinsic resistance to 3GC (which are recommended for calculated antibiotic therapy by the current guidelines), further analysis was performed by including all patients from whose bile samples enterococci were cultured in the cohort of patients with MDR bacteria. In this analysis, only a prior biliary stenting (60% vs. 39%, p = 0.025) remained a significant risk factor for the occurrence of MDR pathogens, including enterococci (S1).

**Table 3:** Risk factors for acute cholangitis with multi-resistant pathogens* in the bile.

	With multi-resistant pathogens* (n = 24)	Without multi-resistant pathogens (N = 59)	P-value
Sex male (n)	20 (83.3%)	35 (59.3%)	**P = 0.043**
Admitted to ICU (n)	2 (8.3%)	8 (13.6%)	P = 0.401
Age (years)	67.5 (61.75; 71.5)	69 (59; 79)	P = 0.527
Length of stay (days)	12.5 (6.75; 21)	8 (6; 15.5)	P = 0.177
Hospital associated (n)	14 (58.3%)	14 (32.2%)	**P = 0.047**
WBC (GPT/l)	8.9 (4.7; 11.5)	10.5 (7.0; 15.1)	**P = 0.014**
CRP (mg/l)	95.7 (67.7; 170.8)	79.9 (34.7; 166.4)	P = 0.241
ALT (μmol/l×s)	0.98 (0.58; 1.59)	1.07 (0.71; 2.12)	P = 0.248
AST (μmol/l×s)	0.93 (0.56; 1.70)	1.19 (0.64; 2.30)	P = 0.490
AP (μmol/l×s)	7.21 (4.21; 9.47)	3.20 (1.99; 5.69)	**P = 0.021**
GGT (μmol/l×s)	11.10 (3,82; 18.18)	5.88 (2.58; 11.1)	**P = 0.040**
Bilirubin (μmol/l)	40.2 (14.5; 98.3)	49.5 (21.0; 121.8)	P = 0.657
Prior ERCP	19 (79.2%)	33 (55.9%)	P = 0.092
Prior biliary stent	18 (75.0%)	26 (44.1%)	**P = 0.025**
Prior papillotomy	19 (79.2%)	32 (54.2%)	P = 0.072
PSC	0	5 (8.5%)	P = 1.000
Choledocholithiasis	10 (41.7%)	36 (61.0%)	P = 0.145
Biliary cancer	7 (29.2%)	7 (11.9%)	P = 0.102
Biliary obstruction at ERCP	19 (79.2%)	36 (61.0%)	P = 0.781
Previous cholecystectomy	12 (50.0%)	24 (40.7%)	P = 0.296
Immunosuppression	8 (33.3%)	8 (13.6%)	P = 0.063
Antibiotics 14 days before admission	10 (41.7%)	9 (15.3%)	**P = 0.012**
Positive blood cultures	14 (58.3%)	14 (23.7%)	**P = 0.019**
Blood cultures with MDR bacteria or fungi**	5 (20.8%)	1 (1.7%)	**P = 0.007**

* ESBL-producing *Enterobacteriaceae* (N = 13), VRE (N = 7) or *Pseudomonas aeruginosa* (N = 4) in the bile.

** ESBL-producing *Enterobacteriaceae* (N = 3), MRSA (N = 1), *Pseudomonas aeruginosa* (N = 1) or *Candida albicans* (N = 1) in the blood.

Multivariate analysis (including sex, nosocomial acquisition, prior BD stenting and antibiotic exposure), performed using backward exclusion, revealed that a prior biliary stenting was the only independent predictor of AC with MDR bacteria (OR = 3.808; 95% CI 1.323–10.960, *P* = 0.013) or AC with enterococci (OR = 3.694; 95% CI 1.408–9.695; *P* = 0.008).

### Course and outcome of AC with MDR bacteria

There were no significant differences in the length of hospital stay of patients with AC by MDR bacteria compared with that of patients without these pathogens (12.5 days vs. 8 days; *P* = 0.18). There were two in-hospital deaths following AC, one involving a patient with AC with Enterococci and *Staphylococcus aureus* and the other a patient with AC with *E*. *coli* and *C*. *albicans*. Notably, patients with biliary MDR bacteria had higher levels of cholestasis, as shown by their alkaline phosphatase (AP) and gamma-glutamyl transpeptidase (GGT) levels, but had lower white-blood-cell counts, indicating less severe inflammation (Table 3). However, patients whose bile cultures were positive for MDR pathogens more often had concomitant positive blood cultures (58% vs. 24%; *P* = 0.019). In addition, bloodstream infection by MDR bacteria or *Candida* spp. was diagnosed significantly more often in patients with MDR pathogens in the bile (21% of patients; 36% of blood culture positive patients) compared with patients without biliary MDR pathogens (2% of patients; 7% of blood culture positive patients) (Table 3).

## Discussion

In this study, we found a high proportion of AC due to enterococci and *E*. *coli* in two German university hospitals, which is consistent with the findings of other recent studies [8,22]. The pathogen profile remained stable over different periods. Due to the polymicrobial culture results and the high rate of MDR bacteria in the bile (24/83; 29%), empiric antibiotic treatment was only successful in covering the resistant microorganisms in only a minority of cases (20/67; 30%). We identified previous biliary stenting as the most relevant independent risk factor for developing AC with MDR bacteria or enterococci, which increased the odds by more than three-fold. These results are consistent with those of a recently published study by Schneider *et al*. [23], in which biliary stenting was identified as the major risk factor for developing AC with 3GC-resistant bacteria. Because biliary stents are generally changed regularly, biliary stenting can be considered a surrogate marker for previous hospitalization, which is known to affect both antibiotic exposure and antibiotic resistance profiles [24]. Indeed, our univariate analysis confirmed that antibiotic exposure and hospitalization are risk factors for MDR bacteria occurring in bile.

A recent prospective German study in 120 patients found a high rate of biliary stent polymicrobial colonization, in 96% of the patients, even in the absence of clinical signs of cholangitis. The most frequently observed pathogens were Enterococci (79.3%) and *Enterobacteriaceae* (73.7%). *Candida* spp. were isolated from 55.9% of the patients [13]. Due to these high rates of bacterial colonization, the authors suggested microbiological testing of explanted biliary stents as a helpful diagnostic procedure to identify the causative pathogens of AC [13]. Schneider *et al*. found that Enterococci (22%) *Klebsiella* spp. (10%) and *Candida* spp. (8%) were the most common organisms colonizing indwelling stents, with a predominance of Gram-positive bacteria and fungi after a short indwelling period and an increasing proportion of Gram-negative bacteria after an indwelling period of more than 60 days [25]. Patients undergoing biliodigestive anastomosis had a higher risk of bacterial and fungal colonization of the BD when undergoing preoperative biliary drainage [26] and had a higher risk of AC with MDR bacteria in the case of preoperative biliary duct stenting [27].

The results of our study argue against the use of 3GC as a first-line antibiotic therapy in patients at risk for MDR-associated AC because they do not cover ESBL-producing *Enterobacteriaceae* or enterococci, which are frequently isolated from the bile of patients with AC. In several studies performed throughout the world, the percentage of MDR bacteria isolated from the bile of patients with AC exceeded 20 to 30% [18], and intrinsically 3GC-resistant enterococci are isolated in 36–74% of AC episodes [8,28,22], particularly those involving patients with underlying biliary disease, e.g., sclerosing cholangitis [8], or a liver transplant [28]. In our study, empiric treatment with 3GC did not cover the isolated pathogens in more than 50% of patients and required the escalation of therapy. Metronidazole was added as part of an empiric combination therapy for 19 patients; however, based on the bile culture analyses, it was dispensable in 18 of these cases. In agreement with Lee *et al*., who found that adding metronidazole to the treatment regimen did not improve the outcome of severe AC [29], it appears reasonable to not recommend adding metronidazole as a first-line therapy if emergency drainage of the bile duct can be performed. Based on our data, replacing 3GC with piperacillin/tazobactam as the empiric antimicrobial therapy would reduce the rate of initially inappropriate treatment for *E*. *coli* infections from 45–56% to 3–29% and would cover *E*. *faecalis* strains (22–44%), as well as anaerobes in the overall cohort, but would likely be insufficient for patients with prior biliary stenting.

Our study has several limitations. First, bile cultures do not discriminate colonization from causative infection and because MDR is often associated with a loss of microbial fitness, one may question the clinical relevance of identifying MDR bacteria in bile. However, our study shows that patients with MDR bacteria in the bile more often have concomitant bloodstream infections in general and bloodstream infections by MDR pathogens or fungi in particular, which supports the notion of providing broad-spectrum antibiotic therapy to at-risk patients. Second, the bile samples were obtained primarily by ERCP and may have been contaminated by the autologous oral and duodenal microflora. Although routine bile sampling during ERCP is not generally recommended by current guidelines, it is widely performed in clinical practice in Germany as it allows the identification of pathogens and their antibiotic susceptibility providing a rationale for antibiotic therapy in patients at risk for MDR pathogens [30,31]. This approach is justified by the arguments that the highest bacterial burden during AC is suspected to be in the biliary system, that blood cultures show a lower sensitivity than bile cultures in AC but–if positive–are in high concordance with the corresponding bile cultures, and that even if duodenal contamination may play a role–the presence of MDR gut bacteria may itself impose a risk factor for ascending AC by MDR. Third, because this was a retrospective study, the selection of empiric antibiotic regimens and the reasons for escalation of therapy were diverse. Fourth, because no significant short-term mortality occurred after AC in our study–even in the absence of antibiotic therapy—there were no hard end-points to prove the failure of antibiotic therapy. Recent studies that could show an association between the microbial bile profile and survival were performed retrospectively and identified MDR bacteria or fungi as risk factors for mortality in patients with malignant biliary obstruction [32], primary sclerosing cholangitis [30] or a liver transplant [28,33]. Whether this association was causative and how it can be addressed by appropriate treatment is unknown. To clarify this important diagnostic question, prospective studies involving appropriate bacterial typing are urgently needed.

Based on the *in vitro* resistance data presented in this study, patients with biliary stents who develop AC should receive empiric therapy covering enterococci and ESBL-producing *Enterobacteriaceae* because these patients are at greater risk for bacteremia. Controlled prospective trials are needed to determine whether improved antibiotic therapy translates into better outcomes for patients with AC.

## Supporting Information

S1 Table Risk factors for infection with multi-resistant pathogens or enterococci*. * ESBL-producing Enterobacteriaceae (N = 13); VRE (N = 7), Pseudomonas aeruginosa (N = 4), MRSA (N = 0) and enterococci (N = 58). ** ESBL-producing Enterobacteriaceae (N = 3), MRSA (N = 1), Pseudomonas aeruginosa (N = 1) or Candida albicans (N = 1) in blood.
(DOCX)

## Abbreviations

3GC: third-generation cephalosporins
AC: acute cholangitis
ALAT: alanine-aminotransferase
ASAT: aspartate-aminotransferase
AP: alkaline phosphatase
ERCP: endoscopic retrograde cholangiopancreatography
ESBL: extended-spectrum beta-lactamase
GGT: gamma glutamyl-transferase
ICU: intensive care unit
JUH: Jena University Hospital
LUH: Leipzig University Hospital
MRSA: methicillin-resistant *Staphylococcus aureus*
MDR: multi-drug resistant
UTI: urinary tract infection
VRE: vancomycin-resistant *Enterococcus*
