# Correction: Risk Factors for Multi-Drug Resistant Pathogens and Failure of Empiric First-Line Therapy in Acute Cholangitis

**DOI:** 10.1371/journal.pone.0172373

**Published:** 2017-02-13

**Authors:** Philipp A. Reuken, Dorian Torres, Michael Baier, Bettina Löffler, Christoph Lübbert, Norman Lippmann, Andreas Stallmach, Tony Bruns

There is an error in affiliation 2 for authors Michael Baier and Bettina Löffler. Affiliation 2 should be: Institute of Medical Microbiology, Jena University Hospital, Jena, Germany.

There is also an error in affiliation 3 for author Christoph Lübbert. Affiliation 3 should be: Division of Infectious Diseases and Tropical Medicine, Department of Gastroenterology and Rheumatology, Leipzig University Hospital, Leipzig, Germany.
