# Supplementary material for: Risk Factors for Multi-Drug Resistant Pathogens and Failure of Empiric First-Line Therapy in Acute Cholangitis
Source: PLoS One. 2017 Jan 11;12(1):e0169900. doi: 10.1371/journal.pone.0169900 (PMC5226732; doi:10.1371/journal.pone.0169900)
Supplement: S1 Table — * ESBL-producing Enterobacteriaceae (N = 13); VRE (N = 7), Pseudomonas aeruginosa (N = 4), MRSA (N = 0) and enterococci (N = 58). ** ESBL-producing Enterobacteriaceae (N = 3), MRSA (N = 1), Pseudomonas aeruginosa (N = 1) or Candida albicans (N = 1) in blood. (DOCX) [file pone.0169900.s001.docx]

**Supplementary Table S1: Risk factors for infection with multi-resistant pathogens or enterococci***

|  | **With multi-resistant pathogens or enterococci (n=55)** | **Without multi-resistant pathogens or enterococci (N=28)** | **P-value** |
| --- | --- | --- | --- |
| Sex male (n) | 42 (76.4%) | 13 (46.4%) | **P=0.013** |
| Admitted to ICU (n) | 7 (12.7%) | 3 (10.7%) | P=1.000 |
| Age (years) | 66 (59.5; 74) | 71.5 (61.75; 82) | P=0.146 |
| Length of stay (days) | 9 (6; 17) | 8 (6; 18.5) | P=0.776 |
| Hospital associated (n) | 22 (40.0%) | 11 (39.3%) | P=1.000 |
| WBC (GPT/l) | 9.65 (7.33; 12.63) | 11.5 (8.45; 17.1) | **P=0.047** |
| CRP (mg/l) | 89.5 (49.1; 173.3) | 68.4 (27.75; 139.25) | P=0.155 |
| ALT (μmol/l×s) | 1.08 (0.61; 1.80) | 0.95 (0.73; 2.76) | P=0.628 |
| AST (μmol/l×s) | 1.15 (0.65; 2.14) | 0.93 (0.59; 1.54) | P=0.417 |
| AP (μmol/l×s) | 5.31 (2.08; 8.36) | 2.99 (2.04; 5.33) | P=0.092 |
| gGT (μmol/l×s) | 8.72 (3.04; 14.63) | 5.59 (2.43; 10.57) | P=0.195 |
| Bilirubin (μmol/l) | 43. (15.0; 108.5) | 51.0 (21.0; 125.5) | P=0.760 |
| Prior ERCP | 40 (72.7%) | 12 (42.9%) | P=0.092 |
| Prior biliary stent | 33 (60.0%) | 11 (39.3%) | **P=0.025** |
| Prior papillotomy | 39 (70.9%) | 12 (42.9%) | P=0.072 |
| PSC | 3 (5.5%) | 2 (7.1%) | P=0.585 |
| Choledocholithiasis | 27 (49.1%) | 19 (67.9%) | **P=0.037** |
| Biliary cancer | 11 (78.6%) | 3 (10.7%) | P=0.171 |
| Biliary obstruction at ERCP | 33 (60.0%) | 22 (78.6%) | P=0.562 |
| Previous cholecystectomy | 27 (49.1%) | 9 (32.1%) | P=0.072 |
| Immunosuppression | 13 (23.6%) | 3 (10.7%) | P=0.090 |
| Antibiotics 14 days before admission | 16 (29.1%) | 3 (10.7%) | P=0.097 |
| Positive blood cultures | 23 (41.8%) | 5 (17.9%) | P=0.110 |
| Blood cultures with MDR bacteria or fungi** | 6 (10.9%) | 0 | P=0.075 |

* ESBL-producing *Enterobacteriaceae* (N=13); VRE (N=7), *Pseudomonas aeruginosa* (N=4), MRSA (N=0) and enterococci (N=58).

** ESBL-producing *Enterobacteriaceae* (N=3), MRSA (N=1), *Pseudomonas aeruginosa* (N=1) or *Candida albicans* (N=1) in blood.
